# Immunodeficiency Hiding in Plain Sight

**DOI:** 10.7759/cureus.27571

**Published:** 2022-08-01

**Authors:** Tyler C Argyle, Adhish Singh, Farhan Abdullah

**Affiliations:** 1 Internal Medicine, Methodist Health System, Dallas, USA

**Keywords:** pancytopenia, splenomegaly, parvovirus, cmv, primary immunodeficiency, hyperigm syndrome

## Abstract

Primary immunodeficiency syndromes encompass a wide variety of inborn and acquired cellular and signaling defects. They are predominantly diagnosed during childhood but can present later into young adulthood depending on the severity, impact, and access to healthcare. Early clues to diagnosis include atypical and severe or recurrent presentations to common pathogens, vaccine failure, and immune lab abnormalities. Despite seemingly obvious characteristics, diagnosis is frequently delayed by months to years at a cost of greatly increased morbidity. Here we present a case of a challenging hyper IgM syndrome diagnosed after seven months and multiple hospitalizations for unique multisystem pathologies.

## Introduction

Inborn error of immunity (IEI), previously known as primary immunodeficiency (PID), is a term that describes a heterogenous array of inherited disorders of both the adaptive and innate immune systems. IEI can occur at nearly every level of immune cell production, from their genetic code to downstream immune products such as antibodies and signaling processes [1]. The effects of these deficiencies are widespread and IEI is now also being implicated in the development of non-infectious complications including autoimmune disorders, lymphoproliferations, and malignancies [1]. Detection of these diseases usually occurs during childhood due to recurrent or severe infections, failure to thrive, and death when the immune defect is significant. Less commonly, IEI is diagnosed in adulthood as timely diagnosis relies on factors such as access to healthcare, proximity to specialists, and availability of relevant lab tests. In this case, we report a 29-year-old patient who presented multiple times throughout a nearly one-year course with diverse organ manifestations of a probable hyper IgM syndrome masquerading as hematologic, infectious and liver diseases.

This article was previously published in the Fall 2020 GME Journal of Methodist Dallas Medical Center.

## Case presentation

**Figure 1:**
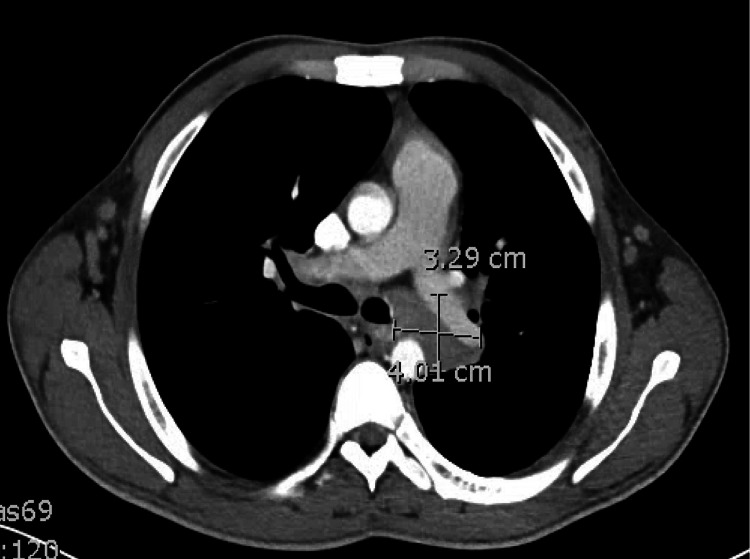
Computerized tomography scan of the chest showing enlarged mediastinal lymph node.

**Figure 2:**
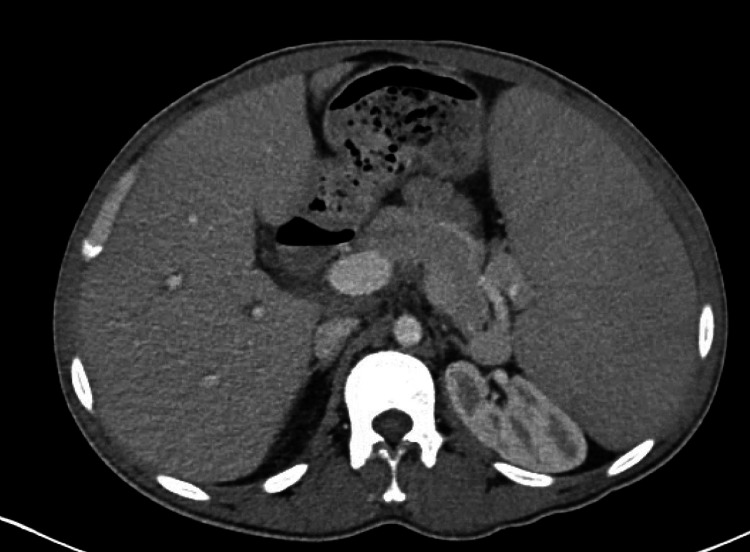
Computerized tomography scan of the abdomen showing massive splenomegaly.

**Figure 3:**
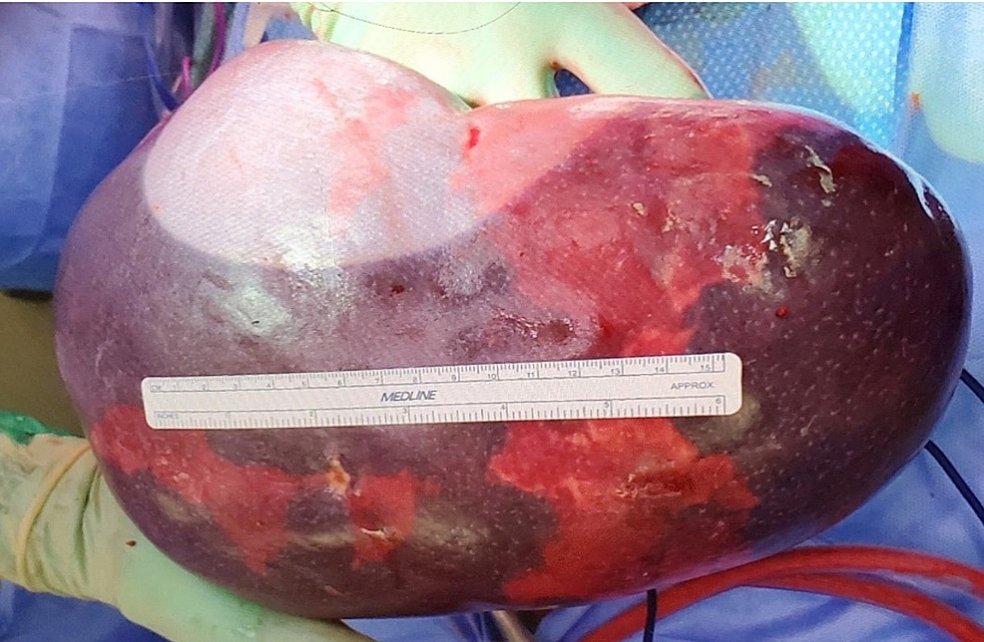
Surgically removed spleen measuring 13 cm x 22 cm and weighing 2150 g

A 29-year-old male with medical history in his adolescence of a “lung infection” that required chest tube placement and hospitalization was sent to the hospital for severe anemia after presenting with fatigue and early satiety to his PCP one day earlier. Further workup revealed pancytopenia and computerized tomography (CT) of the chest and abdomen showed diffuse lymphadenopathy (Figure 1) with mild splenomegaly. Bone marrow biopsy demonstrated pancytopenia, left-shifted maturation, a non-specific pattern of necrosis and an increased T-cell frequency without clonality or hematolymphoid malignancy. Core and excisional biopsies of lymph nodes were highly suspected to demonstrate lymphoma but instead only showed an exuberant reactive process. Cytomegalovirus (CMV) viremia was noted at that time with viral levels below the limit of quantitation, which was felt to be a reactivation by infectious disease and would not account for the current clinical picture. Antinuclear antibody, antineutrophil cytoplasmic autoantibody, rheumatoid factor, erythrocyte sedimentation rate, C3 and C4 complements, anti-cyclic citrullinated peptide (anti-CCP) antibody, and anti double stranded DNA antibody were all normal and a rheumatologic cause was not pursued further. The patient was followed closely in clinic and required multiple blood transfusions in the ensuing two months before being admitted again with accelerated splenic enlargement as well as reduced blood transfusion longevity. A CT scan of the abdomen was notable for massive splenomegaly (Figure 2). Splenic artery embolization and a subsequent splenectomy were performed in which congestive splenomegaly was discovered (Figure 3). Pathologic evaluation of the removed spleen found that it was positive for parvovirus but negative for the stigmata of malignancy. By the time of discharge, the patient was no longer transfusion-dependent and his pancytopenia was improving.

The patient presented to the hospital two months later with new ascites, thrombocytopenia, macrocytosis and transaminitis with elevated alkaline phosphatase concerning for an acute hepatic process. Large-volume paracentesis revealed a low serum-ascites albumin gradient. An ultrasound of the liver showed patent blood vessels and an echocardiogram revealed a normal heart ejection fraction without any structural or functional abnormalities. An acute hepatitis assay was negative though ferritin was noted to be >3,000 at that time. A CT scan of the abdomen showed progression of the lymphadenopathy with new enlarged nodes. A transjugular liver biopsy showed diffuse sinusoidal infiltration with predominantly T lymphocytes and early-stage perisinusoidal fibrosis. Flow cytometry of the liver biopsy sample and a repeat bone marrow biopsy showed B cell proliferation but no clonal component; all samples were parvovirus positive. He was started on diuretics and discharged home but returned to the hospital two days later with worsening ascites, bacterial peritonitis, empyema and bacteremia from *Streptococcus pneumoniae*, against which he had been fully vaccinated at the time of his splenectomy. Due to the patient’s declining hemoglobin level, an esophagogastroduodenoscopy was performed, which revealed portal gastropathy with bleeding ulcers that appeared as stigmata of worsening infiltrative liver disease. A repeat liver biopsy confirmed advancing infiltration with non-clonal T cells and ongoing parvovirus infection. Infectious disease input was requested and a battery of infectious titers yielded positive *Toxoplasma gondii* and *Typansoma cruzi* antibodies in addition to known parvovirus and CMV, though these were all felt to be bystanders to the mysterious and evolving multisystem pathologic process. With a curious array of infectious titers and apparent vaccine failure, attention turned to immune status and a serum immunoglobulin quantification assay revealed that all immunoglobulin levels were severely decreased except for IgM, which was abnormally elevated (Table 1). A diagnosis of probable hyper IgM syndrome was made at that time and the patient was started on intravenous immunoglobulin therapy. His pneumonia resolved and he was discharged with follow-up to the university hospital where further testing revealed normal CD40, CD40L and a *de novo* germline STAT3 gain of function mutation with immunodeficiency and hyper IgM syndrome.

**Table 1:** Immunoglobulin profile of the patient showing severely decreased IgG, most IgG subtypes and IgA levels with a concomitant markedly elevated IgM level.

Immunoglobulin	Value (mg/dL)	Reference Range (mg/dL)
IgG	12	768 - 1632
IgG1	<15	240 - 1118
IgG2	<20	124 - 549
IgG3	3	21 - 134
IgG4	3	1 - 123
IgA	<7	68 - 408
IgM	956	35 - 263

## Discussion

CMV and parvovirus in an otherwise immunocompetent individual would rarely be considered capable of producing diffuse lymphadenopathy, profound pancytopenia, massive splenomegaly, hepatic infiltration or hepatic failure. In our patient, persistent CMV viremia with a viral count below the level of quantitation appeared to be a harmless reactivation, but was likely driving the dramatic pancytopenia and lymphadenopathy with which he presented. The patient’s sequestration and hypersplenism was also most likely due to parvovirus, which has been documented to cause hepatic dysfunction in immunocompromised states [2]; however, the pattern of infiltration and fibrosis documented in this report is unique to those reported thus far. This unusual presentation led to a repeat liver biopsy and further delay in diagnosis. His curious array of positive infectious titers was possibly due to cross reactivity with the hyper IgM state, although quantitative viral loads were not obtained and could have genuinely represented multiple endemic pathogens contracted prior to immigrating with his immunodeficiency.

Our patient’s final presentation of sepsis and empyema likely due to vaccine failure and immune dysregulation presented a more typical mechanistic example of IEI. The delay in diagnosis in this case was around seven months and may represent a more acute and severe course given the failure to thrive and repeated hospital presentations. Regrettably, a key lab study that was lost in the diagnostic milieu, but would likely have hastened diagnosis, was a serum protein electrophoresis showing hypogammaglobulinemia during his first hospitalization. This test was repeated during his fourth hospitalization with similar findings and supported the request for the immunoglobulin quantification assay that revealed the hyper IgM state.

An unusual variable in this case was our patient’s age at diagnosis. IEI is frequently diagnosed at a younger age due to infection of the respiratory tract with bacterial, viral, or even fungal pathogens. However, improved supportive therapies as well as recognition, reporting, and advancements in diagnostic assays are pushing the average age at diagnosis until well past adulthood for some of these diseases [3]. The reasons behind our patient’s age at diagnosis is unclear as he only reported one hospitalization for severe pneumonia and empyema prior to emigrating and immunodeficiency workup is not a typical component of that evaluation.

Multiple countries have begun forming registries to track PID in order to better understand and predict true population distribution [4-8]. Despite these advances, the diagnosis of many immunodeficiency syndromes of clinical significance remains difficult, particularly in the inpatient setting where continuity is limited and fragmentation of care is routine. This setting is a frequent “first contact” for persons suffering from PID and proper diagnosis requires a high index of suspicion to unify what may be multi-system dysregulation. Correct identification and prompt therapy may prevent years of accumulating comorbidities and expensive medical care [9].

## Conclusions

This case demonstrates that the diagnosis of immunodeficiency can be challenging and elusive, often requiring careful review of months to years of prior healthcare presentations in order to expedite the diagnosis. A high index of suspicion must be maintained by the astute clinician, particularly when unusual presentations of illnesses in individuals presumed to be immunocompetent arise. Swift diagnosis can prevent years of accumulating comorbidity and significant healthcare cost.
